# Human seminal plasma hypersensitivity: an underrecognized cause of postcoital allergic symptoms

**DOI:** 10.1097/JW9.0000000000000280

**Published:** 2026-08-05

**Authors:** Kimberly Murdaugh, Wai Wai Mon, Alexis Flen, Mary F. Lee-Wong

**Affiliations:** a New York City Department of Health & Mental Hygiene, New York City, New York; b Department of Medicine, Maimonides Medical Center, New York City, New York; c Department of Pediatrics, Maimonides Medical Center, New York City, New York; d Department of Medicine, Division of Allergy and Immunology, Maimonides Medical Center, Icahn School of Medicine at Mount Sinai, New York City, New York

**Keywords:** allergy, atopy, human seminal plasma hypersensitivity, perimenopause

**What is known about this subject in regard to women and their families?**

- Human seminal plasma hypersensitivity (SPH) is a rare allergic reaction to proteins in seminal fluid that can cause localized vulvovaginal symptoms and, less commonly, systemic allergic manifestations following exposure.
- Fewer than 100 cases have been reported in the literature, although the true prevalence is likely underestimated because of diagnostic delay, symptom overlap with common gynecologic or infectious conditions, and the sensitive nature of discussing postcoital symptoms.
- Management focuses on symptom prevention, patient counseling, and preservation of sexual and reproductive health.
- Consistent condom use is the first-line management strategy and serves as both a diagnostic and therapeutic intervention.
- For women desiring pregnancy or unable to use barrier protection, desensitization strategies may be considered.

**What is new from this article as messages for women and their families?**

- Seminal plasma hypersensitivity can present or be newly recognized in perimenopausal women, particularly in those with underlying atopy.
- Recurrent postcoital vulvovaginal or systemic allergic symptoms that resolve with condom use should prompt consideration of SPH.
- Skin prick testing using a partner’s seminal fluid can support the diagnosis and help distinguish SPH from other causes of vulvovaginal symptoms.
- Recognition of SPH can reduce unnecessary testing, misdiagnosis, and patient distress while facilitating appropriate counseling and shared decision-making regarding sexual activity and family planning.

*Dear Editors*,

Human seminal plasma hypersensitivity (SPH) is a rare allergic reaction to proteins in seminal fluid that can present with localized genital symptoms and/or systemic allergic manifestations following exposure. Fewer than 100 cases have been reported in the literature, although the true prevalence is likely underestimated because of diagnostic delay, overlap with common gynecologic or infectious conditions, and the sensitive nature of symptom disclosure.^1–7^ We report a case of SPH in a perimenopausal woman with atopy, highlighting the importance of clinical suspicion in this population.

A 45-year-old perimenopausal woman with a history of allergic rhinitis and multiple food allergies presented with recurrent abdominal pain, vaginal burning, vulvar erythema, pruritus, and urticarial lesions occurring within 15 minutes of vaginal exposure to seminal fluid. She denied other symptoms of systemic allergic reaction, including respiratory compromise, cardiovascular symptoms, hypotension, or syncope. Symptoms were reproducible and temporally associated with unprotected intercourse. She denied similar symptoms with condom use or periods of abstinence. Evaluation for infectious etiologies and vulvovaginal dermatoses was unrevealing. The patient denied anticipatory anxiety, dyspareunia in the absence of exposure, relationship stressors temporally associated with symptom onset, or symptoms during protected intercourse, arguing against psychogenic sexual dysfunction.

The differential diagnosis for postcoital vulvovaginal symptoms includes vulvovaginal candidiasis, bacterial vaginosis, allergic contact dermatitis to substances such as lubricants or latex, irritant contact dermatitis, vulvodynia, sexually transmitted infections, and psychogenic sexual dysfunction. The reproducible timing of symptoms following unprotected intercourse, resolution with barrier protection, and supportive skin testing findings favored SPH.^1–9^ Initial evaluations for infectious and dermatologic etiologies were unrevealing. Given the consistent timing of symptoms following unprotected intercourse and symptom prevention with barrier protection, SPH was suspected. Seminal fluid was collected by the patient’s partner in a sterile container in a private setting immediately before testing and was used without further processing; spermatozoa were not centrifuged or otherwise removed. Skin prick testing was performed on the volar surface of the forearm using the patient’s partner’s seminal fluid at undiluted and at 1:10, 1:100, and 1:1000 dilutions. Systemic symptoms assessed included urticaria beyond the test site, angioedema, respiratory symptoms, gastrointestinal symptoms, hypotension, and syncope. A burning sensation was reported at the undiluted and 1:10 concentrations, without progression to systemic symptoms. Histamine control produced a 3-mm wheal with a 13-mm flare. Seminal fluid testing demonstrated a 7-mm wheal without flare at the undiluted concentration, a 2-mm wheal without flare at the 1:10 dilution, and no wheal or flare at the 1:100 or 1:1000 dilutions.

In classic immunoglobulin E (IgE)-mediated immediate hypersensitivity reactions, histamine release results in both a wheal, reflecting increased vascular permeability, and a surrounding flare mediated by axon-reflex vasodilation and sensory nerve activation, often accompanied by pruritus.^10^ In this case, histamine control testing produced both a wheal, flare, and pruritus, whereas seminal plasma testing elicited a dose-dependent wheal without associated flare or pruritus, accompanied instead by a burning sensation. This pattern may reflect a localized non-IgE-mediated immune response, a low-level IgE response insufficient to trigger neurogenic flare, or a direct irritant effect of seminal plasma constituents, which include proteolytic enzymes, prostate-specific antigen, and zinc-binding proteins, and have an alkaline pH. While irritant reactions remain part of the differential diagnosis, the reproducibility of symptoms with vulvovaginal exposure, prevention with barrier protection, dose-dependent wheal formation on prick testing, and established literature describing similar localized SPH presentations support the diagnosis.^1,2,4–9^

Two immunologically distinct hypersensitivity reactions have been described: IgE-mediated type I reactions to seminal plasma proteins and type II–like reactions directed against spermatozoa.^4^ Type I reactions most commonly involve prostate-specific antigen, whereas type II–like reactions are associated with antisperm antibodies.^4^ While many cases present early in sexual life, late-onset SPH has been reported and may relate to hormonal changes during perimenopause, alterations in vaginal microbiota, and heightened immune reactivity in individuals with atopy.^1,2,4,6,7^ Changes in seminal plasma composition related to infection, medication use, or dietary factors in the male partner have also been proposed as contributing factors.^1–8^

Management of SPH focuses on symptom prevention, patient counseling, and preservation of sexual and reproductive health.^1,2^ First-line therapy consists of avoidance of allergen exposure through consistent condom use, which is both presumptively diagnostic and therapeutic.^1–3^ For patients desiring pregnancy or those unable to tolerate barrier methods, desensitization strategies may be considered.^1,2,4,7^ Intravaginal graded desensitization using serial dilutions of whole seminal fluid has been shown to be effective in many cases, particularly for localized reactions.^1,2,9^ Subcutaneous immunotherapy with fractionated seminal plasma proteins derived from the patient’s partner has also been described for systemic reactions. Maintenance typically requires regular ongoing low-dose exposure to seminal fluid at a frequency sufficient to sustain immunologic tolerance.^1,2,9^ The patient was counseled on the diagnosis and advised to use consistent barrier protection, which resulted in complete prevention of symptoms. Desensitization options were discussed; however, given adequate symptom control with barrier protection and no current desire for pregnancy during the perimenopausal period, these were deferred.

This case underscores the importance of considering SPH in perimenopausal women presenting with recurrent postcoital vulvovaginal or systemic allergic symptoms, particularly in the setting of atopy. Increased clinician awareness may reduce diagnostic delay and improve patient outcomes.

## Conflicts of interest

None.

## Funding

None.

## Study approval

N/A.

## Author contributions

MFLW, WWM, and AF: Conceived the study, collected clinical data, and approved the final version of the manuscript. KM: Contributed to clinical data interpretation and drafted the manuscript.

## Patient consent

Written informed consent for publication of the patient’s clinical information and accompanying images was obtained from the patient.
